# Artificial Intelligence-Based Decoding of Animal Micro-Expressions: A Review of Methodological Advances and Translational Applications

**DOI:** 10.3390/ani16162578

**Published:** 2026-08-18

**Authors:** Feng Su, Yangzhen Wang, Xiaying Li, Yusheng Wei, Yonglu Tian

**Affiliations:** 1 College of Future Technology, Peking University, Beijing 100871, China; sufeng@pku.edu.cn; 2 Department of Automation, Tsinghua University, Beijing 100084, China; wangyangzhen@163.com; 3 Laboratory Animal Center, School of Life Sciences, Peking University, No. 5 Yiheyuan Road, Haidian District, Beijing 100871, China; lixiaying@pku.edu.cn; 4 IDG/McGovern Institute for Brain Research, Peking University, No. 5 Yiheyuan Road, Haidian District, Beijing 100871, China

**Keywords:** animal micro-expression, artificial intelligence, facial action coding system, animal welfare, deep learning

## Abstract

Animal micro-expressions constitute transient behavioral windows that link internal states to externally observable signals, while artificial intelligence (AI) serves as the critical bridge that transforms these windows into measurable, interpretable, and applicable scientific tools. Rather than imposing a human-centered lexicon of expressions, AI-driven decoding aims to develop biologically grounded and increasingly comparable behavioral biomarkers that link computable facial dynamics to internal states; however, a validated universal cross-species framework has not yet been established. This review summarizes the common behavioral characteristics of animal micro-expressions, their cross-species expressive forms, and functional differences; systematically outlines the methodological spectrum through which AI captures, encodes, recognizes, and interprets these brief yet complex signals; and finally discusses the expanded applications of AI plus micro-expression analysis in basic research, clinical diagnosis, and animal welfare governance, thereby promoting a paradigm shift in the decoding of animal micro-expressions.

## Simple Summary

Animals can show brief changes in the face when they are in pain, frightened, stressed, interested, or comfortable. These changes are often too fast or subtle for people to notice consistently, which can make it difficult to assess an animal’s condition without disturbing it. This review explains how computer-based video analysis can help detect and describe these small facial movements in animals. It considers evidence from companion animals, farm animals, laboratory animals, and primates, and discusses how facial signals vary among species. The review also describes practical uses, including earlier recognition of pain or illness, monitoring recovery after treatment, improving the care of animals used in research, and supporting welfare monitoring on farms. Important challenges remain, such as collecting reliable labelled video data and ensuring that computer systems work well across breeds, species, and real-world settings. Used carefully alongside veterinary and behavioural expertise, these tools could make animal assessment more objective, timely, and welfare-focused.

## 1. Introduction

Facial movements provide a rapid and nonverbal channel through which animals express internal biological and affective states. Among these movements, micro-expressions refer to transient, subtle, and often involuntary facial changes that may occur before overt behavioral responses become apparent. These signals are increasingly recognized as sensitive indicators of pain, stress, fear, anxiety, positive affect, social intention, and physiological disturbance [1,2,3]. The Facial Action Coding System (FACS) has provided a foundation for objectively describing human facial movements through anatomically defined action units [4,5]. Its extension to non-human species, including ChimpFACS for chimpanzees [6], MaqFACS for macaques [7], EquiFACS for horses [8,9], DogFACS for dogs [10,11] and CatFACS for cats [12,13], has enabled systematic coding of species-specific facial movements. These frameworks have shifted animal expression research from subjective human interpretation toward anatomically grounded and reproducible behavioral quantification.

However, manual annotation of animal facial movements remains time-consuming, observer-dependent, and difficult to scale. Artificial intelligence (AI) offers a powerful solution by enabling high-throughput analysis of facial dynamics. AI-based methods can detect animal faces, localize facial landmarks, estimate pose, classify expression states and integrate facial information with physiological or environmental data. This review aims to synthesize current knowledge on animal micro-expressions and their AI-based decoding. We first define the key behavioral properties of animal micro-expressions. We then summarize cross-species differences in facial expression systems, followed by an overview of major AI methods used for micro-expression analysis. Finally, we discuss translational applications and future directions, with particular emphasis on animal models and experimental medicine.

## 2. Review Methodology

This review was designed as a narrative synthesis supported by a structured literature search and two-stage screening. The scope covered image classification, landmark and pose analysis, temporal modeling, and multimodal fusion, together with applications in basic research, veterinary care, and animal welfare.

PubMed was used as the core source for a reproducible literature search, which was rerun and verified on 31 May 2026. The search returned 249 records. The exact PubMed query was: (“facial action coding system”[Title/Abstract] OR “grimace scale”[Title/Abstract] OR (“facial”[Title/Abstract] AND (“dog”[Title/Abstract] OR “canine”[Title/Abstract] OR “cat”[Title/Abstract] OR “feline”[Title/Abstract] OR “horse”[Title/Abstract] OR “equine”[Title/Abstract] OR “pig”[Title/Abstract] OR “porcine”[Title/Abstract] OR “cattle”[Title/Abstract] OR “bovine”[Title/Abstract] OR “sheep”[Title/Abstract] OR “goat”[Title/Abstract] OR “primate”[Title/Abstract] OR “macaque”[Title/Abstract] OR “mouse”[Title/Abstract] OR “mice”[Title/Abstract] OR “rat”[Title/Abstract] OR “rodent”[Title/Abstract]))) AND (“artificial intelligence”[Title/Abstract] OR “machine learning”[Title/Abstract] OR “deep learning”[Title/Abstract] OR “computer vision”[Title/Abstract] OR “automatic”[Title/Abstract] OR “automated”[Title/Abstract] OR “classifier”[Title/Abstract] OR “landmark detection”[Title/Abstract] OR “pose estimation”[Title/Abstract]) AND 1900/01/01:2026/05/31[Date—Publication]. Web of Science, Scopus, and IEEE Xplore were used for targeted supplementary searches combining the same animal/species, facial-expression/FACS, and AI/method concept blocks, with syntax adapted to each platform. Google Scholar was used only for supplementary discovery and citation tracing rather than as an exhaustive search source.

During revision, the search results and screening decisions were rechecked. Two authors independently screened titles and abstracts and subsequently assessed potentially eligible articles in full text against the inclusion and exclusion criteria. Disagreements at either stage were resolved through discussion and, when necessary, adjudication by a third author. Reference lists of included papers and key reviews were checked for additional relevant studies. Screening was relevance-based and iterative, consistent with the narrative purpose of the review.

Studies were retained if they addressed at least one of the following: (1) AI-based detection, landmark localization, expression classification, or temporal analysis of animal faces; (2) FACS-based coding of facial movements; (3) associations between facial signals and physiological or affective states; or (4) validation of AI systems in veterinary, laboratory, or livestock settings. Publications were excluded when they were duplicate reports, did not concern animal facial signals or a technically transferable method, lacked sufficient methodological detail to determine the data source, analytical approach, or relevance to the review, or consisted only of conference abstracts, editorials, or other non-peer-reviewed material without adequate technical information. Human-only studies were used only for methodological context and were treated as cross-domain evidence rather than direct animal validation.

Studies were appraised qualitatively for clarity of species and setting, sample or dataset description, acquisition and annotation procedures, subject-independent or external validation, appropriateness of performance metrics and baselines, reporting of limitations, and reproducibility. These criteria were used to weigh the strength and transferability of evidence during synthesis. Studies with partial reporting, restricted settings, or no external validation were not automatically discarded if they remained informative; their limitations were considered explicitly and conclusions were qualified accordingly.

Given the substantial heterogeneity in species, imaging modalities, model architectures, annotation schemes, and evaluation metrics, a quantitative meta-analysis was not considered appropriate. Findings were therefore synthesized qualitatively through thematic grouping by methodological type and application domain.

This study did not involve animal experimentation or primary data collection; therefore, ethical approval was not required. Large language models (LLMs) were only used to assist in drafting initial schematic figures based on conceptual models developed by the authors. All scientific content was verified, edited, and finalized by the authors. No generative AI tools were used for primary data generation or analysis.

## 3. Results

### 3.1. Core Characteristics of Animal Micro-Expressions

Animal micro-expressions represent coordinated physiological and behavioral responses that provide visible access to otherwise hidden internal states. Across species, they share several key characteristics.

**Transience.** Micro-expressions are temporally brief and often difficult to detect through direct visual observation. In humans, micro-expressions typically last approximately 300 ms [14], while equine facial micro-expressions may last less than 500 ms [9]. This high temporal resolution requires high-speed video acquisition, frame-by-frame annotation, and computational models capable of capturing rapid temporal changes [15]. The transient nature of micro-expressions is therefore one of the primary reasons AI-based analysis is essential.

**Diversity and Combinatorial Complexity.** Animal micro-expressions are not isolated movements but combinations of multiple local facial actions involving the eyes, eyelids, orbital region, ears, nose, muzzle, mouth, lips, whiskers, cheeks, and head posture. FACS-based studies have shown that cats, dogs, horses, and primates generate diverse facial configurations through combinations of action units [8,9,16,17]. This combinatorial complexity creates a large expression space, making manual interpretation difficult but computational representation feasible.

**Dynamic Temporal Structure.** Micro-expressions include rapid onset, peak, transition, and offset phases [14,18]. Their meaning often depends not only on a single facial configuration but also on the trajectory of facial changes over time. The same animal may show different facial patterns depending on stimulus intensity, duration, social partner, and preceding behavioral context [10]. Therefore, micro-expression research should move beyond static image classification toward dynamic modeling of facial motion and temporal evolution [18,19].

**Species Specificity.** Species differ substantially in facial musculature, craniofacial structure, fur coverage, ear mobility, whisker function, and reliance on visual communication [3,4]. For example, dogs display facial signals that are highly readable to humans due to domestication and long-term human–dog coevolution [16]. Primates possess complex facial repertoires associated with social hierarchy and affiliative behavior [17]. Rodents, by contrast, show relatively subtle facial changes that are especially informative in pain and stress research [20,21]. Thus, AI models must be designed with species-specific anatomy and behavioral ecology in mind.

**Mapping to Physiological and Health States.** Facial micro-expressions can reflect physiological conditions such as pain, inflammation, discomfort, neural injury and cognitive load [22]. These facial signals often co-occur with changes in heart rate, respiration, pupil size, muscle tone, and neural activity [3]. Pain-related facial changes, including orbital tightening, facial tension, and altered muzzle configuration, have been reported across several species [21,22]. This makes micro-expression analysis especially valuable for non-invasive health monitoring.

**Mapping to Emotion and Intention.** Micro-expressions are also associated with affective and motivational states, including fear, anxiety, excitement, aggression, affiliation, avoidance, and anticipation [2,23,24,25]. Importantly, subtle facial movements may emerge before large-scale postural or locomotor changes, offering an early behavioral indicator of internal state transitions [3]. This property is particularly important for early warning systems in veterinary care, laboratory animal monitoring, and welfare assessment.

**Context Dependence.** Animal micro-expressions are not fixed outputs but context-dependent signals. In human–dog interactions, canine facial expressions differ between positive conditions, such as play or being called by name, and negative conditions, such as separation or exposure to strangers [11,26]. Micro-expressions may also be modulated by human attention [27]; thus micro-expression interpretation must integrate environmental stimuli, social partners, prior experience, and behavioral context [28].

This synthesis of core characteristics reveals several overarching principles that not only define the nature of animal micro-expressions but also fundamentally shape the methodological landscape for AI-driven analysis. First, the intrinsic properties of transience, combinatorial complexity, and dynamic temporal structure collectively render manual annotation not only labor-intensive but cognitively intractable—the human visual system is ill-equipped to reliably detect, let alone quantify, rapid multi-region facial changes occurring at millisecond precision. This perceptual bottleneck positions AI not merely as a convenient enhancement but as an indispensable enabler for objective, high-throughput behavioral phenotyping. Second, the confluence of species-specific anatomy and context-dependent signaling highlights a core design tension: AI architectures must balance the pursuit of generalizable, cross-species frameworks with the imperative for species-adapted feature extraction that respects distinct craniofacial morphology, fur coverage, ear mobility, and whisker function. Third, the dual mapping of micro-expressions onto both physiological states (e.g., pain, inflammation) and affective states (e.g., fear, anticipation, affiliation) establishes facial signals as a convergent output channel for multiple internal systems. This convergence is analytically powerful—offering a rich, multidimensional window into animal wellbeing—yet simultaneously poses a profound decoding challenge, as overlapping or co-occurring internal states may produce confounded facial patterns requiring contextual disambiguation. Taken together, these principles translate directly into four methodological imperatives for AI system design: (i) millisecond-scale temporal modeling to capture rapid dynamics; (ii) multi-region combinatorial encoding to represent coordinated action units; (iii) species-aware feature learning to accommodate anatomical diversity; and (iv) contextual integration to resolve state ambiguity. No single existing approach fully satisfies all four requirements, pointing toward integrated multimodal frameworks that combine facial analysis with physiological, environmental, and behavioral context as the necessary next frontier for the field.

### 3.2. Cross-Species Patterns of Animal Micro-Expressions

**Figure 1:**
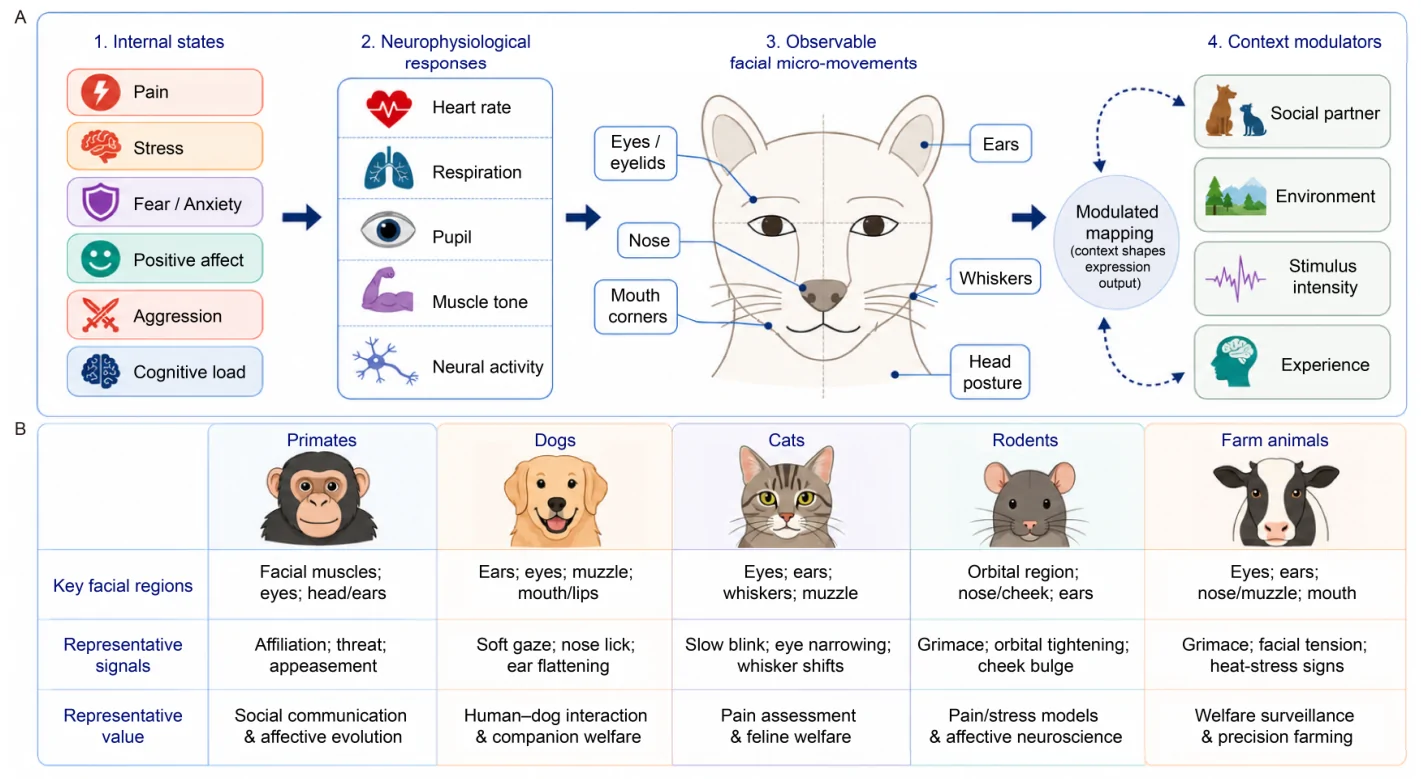
**Cross-species phenotypic atlas of animal micro-expressions.** (**A**) Animal micro-expressions serve as external signals of internal states such as pain, stress, fear, positive emotion, aggressive tendency, and cognitive load. These states are accompanied by neurophysiological changes including heart rate, respiration, pupil size, muscle tone, and neural activity, and are expressed through facial cues involving the eyes/eyelids/orbital region, ears, nose/muzzle, mouth corners/lips, whiskers/cheeks, and head posture. Their interpretation is modulated by contextual factors such as social partners, environment, stimulus intensity, and prior experience. (**B**) Micro-expressions differ across species in their anatomical basis, typical signals, and functional significance. Primates, dogs, cats, rodents, and farm animals are respectively of representative value in contexts such as social communication, human–pet interaction, pain and welfare assessment, and experimental phenotyping.

Although facial micro-expressions are broadly present across mammals, their anatomical basis, expressive form, functional significance, and human readability differ across species [4,23]. Therefore, research on animal micro-expressions must seek both commonality and specificity: it should establish a cross-species framework for comparable state representation while fully characterizing species-specific micro-expressive patterns (Figure 1).

**Primates: Social Complexity and Evolutionary Relevance.** Primates possess some of the most complex facial expression systems among mammals. Their facial movements are closely linked to dominance, alliance formation, conflict, appeasement, affiliation, and social communication [6,7,17,29]. The coordination of facial, head, and ear movements makes primates particularly suitable for video-based tracking and computational analysis [30]. Because primates share important homologies with humans in facial control and emotional neural circuits, they serve as critical models for studying the evolution of affective expression and social emotion [31].

**Canids: Highly Readable Dynamic Social Signals.** Dogs have a rich facial expression repertoire and can produce numerous combinations of facial movements [16]. Typical affiliative signals include forward ear orientation, relaxed eyes, soft gaze, and relaxed mouth configuration. Stress-related signals may include nose licking, ears flattened against the head, lip retraction, and increased facial tension. Fear or anxiety may be accompanied by pupillary changes and tightened facial musculature [10]. Due to human–dog coevolution, canine facial expressions are highly relevant for interspecies communication, companion animal welfare, and affective computing [11,32].

**Felids: Subtle but Information-Dense Facial Signals.** Cats display facial expressions that are often subtle, low in amplitude, and difficult for humans to interpret accurately. Relaxed or affiliative states may involve slow blinking, partial eye closure, forward whiskers, and relaxed mouth corners, whereas aggression or vigilance may be associated with flattened ears, pupillary changes, and facial tension. Pain-related facial changes may include orbital tightening, altered muzzle shape, increased nose-to-mouth distance, and changes in whisker position [12,13]. Because human recognition of feline expressions is often limited and susceptible to anthropomorphic bias [32], AI-assisted analysis may substantially improve feline pain assessment and welfare monitoring [33,34].

**Rodents: Experimental Models for Pain, Stress, and Affective Neuroscience.** Rodents have relatively simple but experimentally valuable facial expression systems [20,21]. Facial grimace scales have been widely used for pain assessment, and facial features have also been linked to fear, anxiety, depression-like states, and responses to pharmacological or neural circuit manipulations [21,35]. Rodents are particularly suitable for establishing causal relationships among stimulus exposure, neural activity, facial expression, and behavioral state under controlled experimental conditions. AI-based high-throughput analysis can enhance reproducibility and reduce observer bias in laboratory animal research [36,37].

**Farm Animals: Welfare Monitoring and Precision Livestock Farming.** Farm animals, including cattle, sheep, pigs, and horses, show facial changes associated with pain, stress, disease, and environmental challenge [38,39,40]. For example, automated facial analysis of calves can support early detection of welfare problems, heat stress, injury, illness, or abnormal recovery [41,42]. In large-scale farming systems, AI-driven facial recognition and expression decoding may contribute to continuous welfare surveillance, early warning, and precision management [40,43,44]. Such systems are particularly valuable where individual-level manual observation is impractical.

Taken together, these cross-species comparisons reveal three overarching patterns with direct implications for AI system design. First, convergent patterns, such as phylogenetically conserved pain indicators across rodents, felids, canids, equids, and primates, support cross-species transfer learning and foundation models that pool data from multiple taxa to enhance performance in data-scarce species. Second, divergent patterns in social-affective signaling, where the same configuration can carry opposite meanings and informative facial regions vary (ears in horses and dogs, whiskers in cats and rodents, brows in primates), preclude a one-size-fits-all architecture. Instead, modular pipelines can combine shared low-level encoders with animal-group-aware pathways, species-adapted anatomical heads, and state-calibration modules, balancing generalizability with anatomical fidelity. Third, human readability varies markedly, with dog signals being more intelligible than feline or rodent expressions. This variability affects annotation reliability and supervised learning, necessitating quality control and reduced dependence on human labels through semi-supervised or unsupervised approaches that leverage temporal or multimodal signals. Collectively, these three insights argue for a dual-track development strategy: (i) building robust, generalizable models that exploit convergent pain-related phenotypes across species; and (ii) engineering species-optimized modules that respect divergent expressive repertoires and variable label reliability, while integrating contextual and multimodal information to resolve state ambiguity. This balanced approach ensures both scientific validity and translational utility, moving the field beyond generic technical pipelines toward biologically informed, application-ready AI systems.

### 3.3. AI-Based Methods for Animal Micro-Expression Analysis

**Figure 2:**
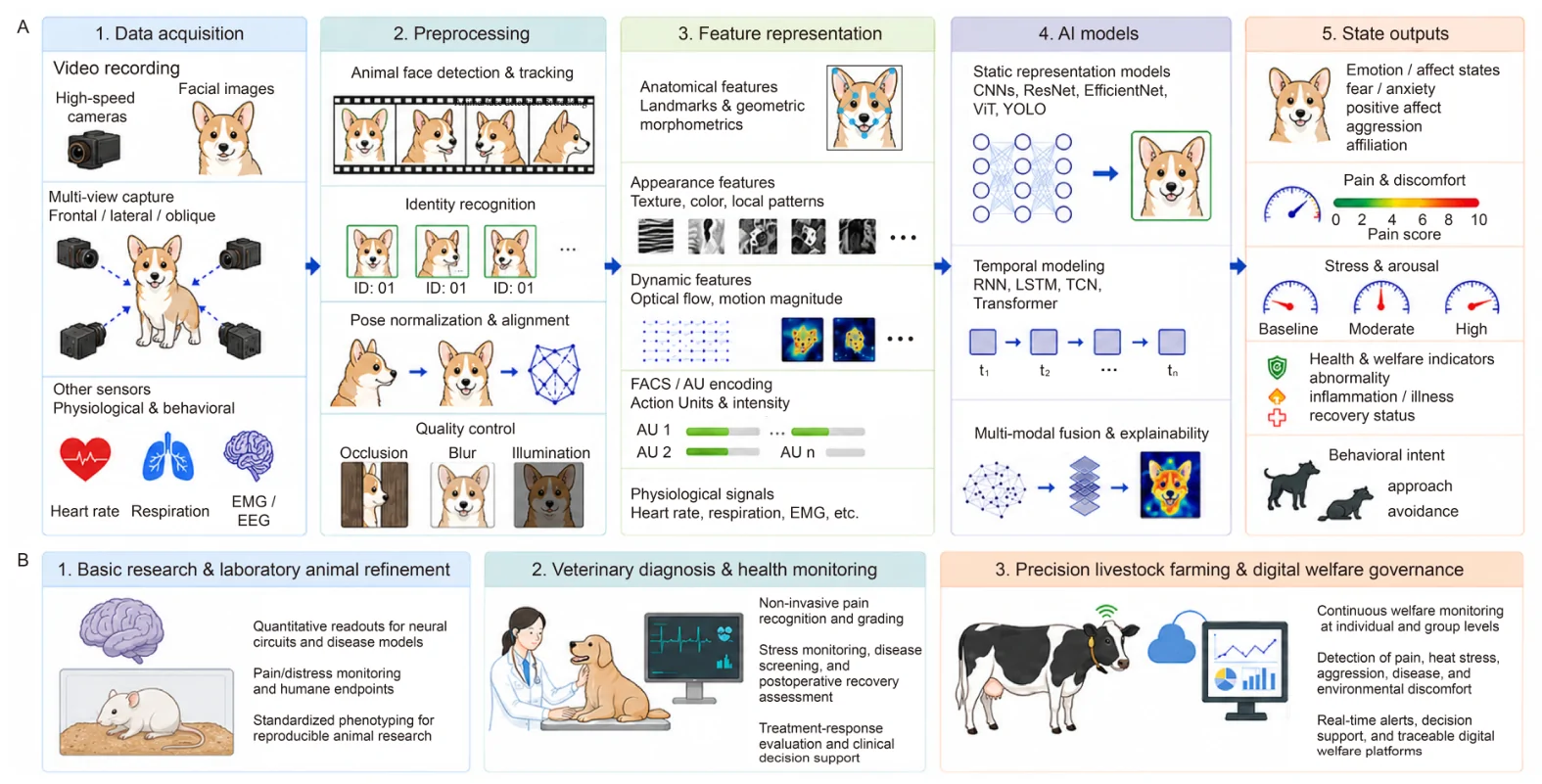
**Technical workflow and application scenarios for AI-based decoding of animal micro-expressions.** (**A**) AI-based micro-expression decoding includes data acquisition, preprocessing, feature representation, model learning, and state output. Input data may include facial images, video sequences, multi-angle recordings, and optional physiological signals. These are followed by face detection, individual identification, pose normalization, and quality control, after which anatomical landmarks, appearance textures, dynamic features, FACS codes, and physiological features are extracted. Finally, image models, temporal models, and multimodal fusion models are used to output emotional states, pain, stress, health and welfare indicators, and behavioral intentions. (**B**) AI-based animal micro-expression analysis can be applied to basic neurobehavioral research, clinical diagnosis and health monitoring, companion animal welfare, precision livestock farming, laboratory animal management, and digital animal welfare governance. Colors are used solely to distinguish workflow stages and application settings and do not encode quantitative values.

AI provides a methodological framework for transforming brief and complex facial signals into quantifiable behavioral biomarkers. Current approaches can be broadly grouped into image classification, anatomical pose estimation, dynamic temporal modeling, multimodal fusion, and emerging foundation-model, vision-language, and generative paradigms. Figure 2 summarizes the end-to-end analytical workflow, with Table 1 and Table 2 comparing representative studies and these method families, respectively.

Table 1 summarizes representative animal studies across species, target states, inputs, and analytical approaches, whereas Table 2 provides a critical comparison of the five major method families, including their data requirements, susceptibility to species differences, and deployment suitability.

#### 3.3.1. Image-Based Micro-Expression Classification

Image classification aims to identify affective or physiological states from single-frame facial images. This task is usually formulated as supervised learning, in which convolutional neural networks and related deep learning models are trained on labeled datasets to map visual facial features to target states [30]. Convolutional neural network (CNN)-based methods have been used to classify affective states such as pleasure, disgust, and fear in mice [37], identify pain- and stress-related facial features in horses [45,46], and detect social facial expressions in equine interactions [9].

Object detection models such as You Only Look Once (YOLO) combined with CNN classifiers have been applied to recognize pig facial expressions under heat stress [47], and to support real-time tracking and classification of canine facial states such as excitement or calmness [48]. Integration of AI models with FACS can further improve interpretability by linking model predictions to anatomically meaningful facial movements [49].

Despite their utility, image-based approaches have limitations. They are sensitive to dataset scale, annotation quality, lighting conditions, pose variation, fur occlusion, individual differences, and domain shifts across experimental environments. Moreover, static images may fail to capture the temporal dynamics that define micro-expressions.

#### 3.3.2. Anatomical Landmark Detection and Pose Estimation

Anatomical pose estimation identifies facial landmarks such as the eye corners, nose tip, mouth corners, whisker pads, and ear bases. These landmarks provide structured geometric representations of facial morphology and movement. Automated landmark detection systems have been developed for cats, dogs, and other animals, allowing extraction of key points across the eyes, ears, nose, mouth, and muzzle [13,34,50].

Landmark-based analysis enables geometric morphometrics, in which relative distances, angles, and deformations among landmarks are used to quantify facial changes in cats and dogs across states [33,51,52,53]. For example, eye narrowing, whisker position, muzzle tension, and ear orientation can be converted into measurable features associated with pain or affective state [54]. Compared with purely appearance-based models, landmark-based methods are more interpretable and can be more directly linked to anatomical action units.

However, landmark detection in animals remains challenging. Species differ in craniofacial structure, facial mobility, fur patterns, and visible landmarks. High-quality annotation is labor-intensive, and models trained on one species or breed may not generalize well to another. Cross-species systems such as AnyFace++ demonstrate the potential of unified multi-task learning for human and animal face analysis, but further validation is required in biological and clinical contexts [55].

#### 3.3.3. Dynamic Temporal Modeling

Because micro-expressions unfold over time, temporal modeling is essential for accurate decoding. Recurrent neural networks (RNN), long short-term memory networks (LSTM), temporal convolutional networks (TCN), 3D convolutional networks, and transformer-based video models can capture temporal dependencies and dynamic facial trajectories. Hybrid CNN–LSTM architectures can jointly model spatial features and temporal evolution [56,57,58], while LSTM and video transformer architectures may better capture global spatiotemporal patterns [18,59].

Recent advances in video Transformers and masked-video pretraining provide reusable temporal representations for animal video analysis [60,61]. In animal studies, temporal models have already been applied to behavior recognition, including the identification of sleep and feeding behaviors in horses from continuous video sequences [62]. For affective or pain-related states, video-based models may capture subtle changes such as twitching, tremor, muscle tension, and transient facial contractions that static images cannot detect. Future systems should therefore prioritize temporally dense video annotation and sequence-level state prediction.

Recent video Transformers extend temporal modeling beyond recurrent or local convolutional operations by applying space-time self-attention across frame patches. Architectures such as TimeSformer model long-range dependencies through factorized spatial and temporal attention, while masked-video pretraining methods such as VideoMAE learn reusable spatiotemporal representations from unlabeled video [60,61]. For animal micro-expression analysis, these approaches could capture distributed, low-amplitude facial changes whose meaning depends on longer behavioral context.

Practical use will nevertheless require domain-aware pretraining and careful validation. A feasible strategy is to pretrain on large volumes of unlabeled, species-specific video using masked reconstruction, temporal consistency, or contrastive objectives, then fine-tune on smaller frame- or sequence-level labeled datasets. Subject-exclusive and cross-site testing are essential because self-supervision reduces label demand but does not eliminate species, breed, camera, or environment shifts.

#### 3.3.4. Multimodal Fusion

Micro-expressions rarely occur in isolation. They are often accompanied by changes in posture, locomotion, vocalization, heart rate, respiration, pupil size, body temperature, and neural activity. Multimodal fusion can integrate facial video with physiological, behavioral, environmental, and neural data to improve decoding accuracy and biological interpretability [63].

For example, combining facial landmarks with heart rate and respiration may improve stress detection, whereas integrating facial dynamics with neural recording may reveal circuit-level mechanisms underlying affective states. In livestock systems, micro-expression analysis may be combined with thermal imaging, accelerometers, feeding records, and environmental sensors. In laboratory animals, AI-based facial analysis can be integrated with optogenetics, calcium imaging, EEG, EMG, or pharmacological manipulation to establish mechanistic links between neural activity and behavioral output [3].

Fusion can occur at three levels. Early fusion concatenates synchronized facial, physiological, and contextual features and are efficient when all modalities are consistently available. Intermediate fusion uses attention or gated shared representations to model cross-modal interactions; such architectures are promising for combining subtle facial motion with temporally aligned physiological signals [63]. Late or decision-level fusion combines independently trained modality-specific predictions and is usually easier to deploy when sensors are asynchronous or intermittently missing. Existing animal evidence supports the value of combining facial dynamics with neural recordings in controlled laboratory studies [20] and of integrating vision with thermal, wearable, and environmental sensors in livestock systems [40,43], but direct head-to-head comparisons remain scarce. Consequently, no fusion level can yet be considered universally superior; external validation, calibration, and robustness to missing modalities should guide selection.

#### 3.3.5. Foundation Models, Vision-Language Models, and Generative Data Augmentation

More broadly, AI-based monitoring has been proposed for animal health and welfare applications [64]. Foundation models and vision-language models extend task-specific pipelines by reusing representations learned from large image, video, or image-text corpora. Pretrained animal models such as SuperAnimal demonstrate that pooled multi-species data can support zero-shot pose estimation and data-efficient adaptation [65]. In broader animal-behavior analysis, vision-language foundation models have also enabled text-defined zero-shot classification without task-specific training [66]. These capabilities may reduce annotation requirements and support open-vocabulary querying, but behavior-level transfer does not by itself establish reliable decoding of subtle facial affects.

Large vision-language models can combine visual inputs with natural-language prompts, descriptions of facial action units, or contextual metadata, potentially supporting few-shot adaptation and interpretable hypothesis generation. However, current animal-affect evidence remains preliminary. In dog emotion recognition, general-purpose LVLMs achieved moderate performance on web-sourced images but fell to near-chance performance on experimentally grounded cropped-face data and were strongly influenced by background context [67]. Accordingly, zero-shot outputs should be treated as hypotheses rather than biological ground truth. Species-specific fine-tuning, temporal inputs, anatomically constrained prompts, uncertainty reporting, and subject-exclusive, cross-breed, and external validation remain necessary.

Generative AI can also support data augmentation by producing or rendering rare poses, viewpoints, occlusions, and environmental conditions. Synthetic animal data have improved pose-related learning under limited real annotation, particularly when combined with consistency constraints and unlabeled real images [68]. For micro-expression applications, synthetic samples should preserve species-specific anatomy and temporal plausibility, be screened by domain experts, and never replace evaluation on independent real data. Generated expressions should not be assigned internal-state labels solely from appearance, because this would reproduce rather than solve the ground-truth problem.

#### 3.3.6. Synthesis and Outlook on AI-Based Methods

The past decade has seen significant advances in AI-driven animal micro-expression analysis. Image-based classifiers now reliably detect pain, stress, and affective states across multiple species from single-frame inputs. Landmark detection pipelines provide anatomically interpretable quantification of facial morphology and movement. Temporal models capture the rapid onset, peak, and offset dynamics that define true micro-expressions. Additionally, multimodal frameworks are beginning to integrate facial signals with physiological and environmental data. Emerging foundation and vision-language models offer reusable representations, text-guided querying, and zero- or few-shot transfer, while generative methods can augment rare conditions; however, animal-affect evidence remains preliminary and requires species-specific validation. These five methodological families, both individually and in combination, have transformed micro-expressions from fleeting, observer-dependent impressions into computationally tractable, quantifiable behavioral biomarkers. Moreover, this technical progress spans a remarkable taxonomic breadth—from primates and canids to felids, rodents, equids, and farm animals—with systems increasingly tailored to species-specific anatomy and expressive repertoires.

Looking ahead, two strategic priorities deserve emphasis. First, decoding animal micro-expressions is inherently a systems-level challenge: effective implementation requires integrated pipelines that coordinate detection, tracking, temporal modeling, and contextual fusion within unified architectures. This approach moves beyond isolated benchmark optimization toward multi-task systems capable of simultaneous expression classification, intensity estimation, and state inference. Second, cross-species model transfer remains underutilized; phylogenetically conserved pain indicators suggest significant opportunities for transfer learning across taxa. Such transfer would improve performance in data-sparse species, enhance training efficiency, and enable direct comparative studies of micro-expression homology and divergence. These directions call for a shift from proliferating isolated species-specific solutions toward reusable, generalizable AI frameworks that support both fundamental comparative research and applied welfare monitoring.

Method selection should therefore be problem-driven rather than accuracy-driven. In small labeled datasets, landmark/geometric models or pretrained static encoders are generally more defensible because they use stronger anatomical priors and are easier to interpret. End-to-end temporal and multimodal models can represent richer state dynamics but require more subjects, denser annotation, synchronized inputs, and stronger regularization. Appearance classifiers are especially sensitive to background, coat, breed, and lighting shifts; landmark models are sensitive to anatomical definitions and occlusion; temporal models to frame rate and sequence length; and multimodal systems to sensor synchronization and missing data. For practical deployment, lightweight image or landmark pipelines are currently the most feasible for triage or edge inference, whereas temporal and multimodal systems are better suited to high-value clinical or controlled research settings. These trade-offs are summarized in Table 2.

### 3.4. Translational Applications of AI Plus Micro-Expressions

#### 3.4.1. State Visualization in Basic and Translational Research

AI-based micro-expression analysis can serve as a “state visualization tool” for experimental medicine and behavioral neuroscience. By converting subtle facial movements into quantitative features, it provides objective phenotypic readouts of emotional and physiological states [30]. This is particularly valuable in animal models of pain, anxiety, depression, neurodegeneration, inflammation, and stress-related disorders. In mechanistic studies, micro-expression decoding can be used to assess behavioral consequences of neural circuit manipulation, pharmacological intervention, genetic modification, or disease modeling [3]. For instance, after optogenetic stimulation or chemogenetic inhibition of specific neural pathways, AI-based facial analysis may provide fine-grained behavioral indicators of affective state changes. Such approaches can strengthen the connection between neural mechanisms and observable behavioral phenotypes.

#### 3.4.2. Nonverbal Assessment in Veterinary Diagnosis and Health Monitoring

AI-assisted micro-expression analysis offers a non-invasive interface for clinical evaluation. It may support pain recognition, pain grading, stress monitoring, disease screening, postoperative recovery assessment, and treatment response evaluation. Facial pain scales have already demonstrated value in species such as cats, sheep, pigs, horses, and rodents [16,29,54]. Automated systems could assist veterinarians by continuously monitoring animals in clinics, shelters, farms, and home environments. Early detection of discomfort, inflammation, abnormal recovery, or behavioral depression may improve clinical decision-making and enable timely intervention [41,64].

#### 3.4.3. Laboratory Animal Welfare and Refinement of Animal Models

In experimental medicine, animal models are indispensable, but ethical standards require refinement, reduction, and replacement wherever possible. AI-based micro-expression analysis can contribute to refinement by enabling more sensitive and objective monitoring of pain and distress. Automated detection of facial changes may reduce reliance on coarse behavioral endpoints and allow earlier humane intervention. For laboratory rodents, high-throughput facial analysis can improve welfare surveillance in disease models, surgical recovery, drug testing, and neurobehavioral experiments. It may also enhance reproducibility by reducing observer bias and standardizing behavioral phenotyping across laboratories.

#### 3.4.4. Precision Livestock Farming and Digital Welfare Governance

In livestock production, scalable AI systems can support continuous monitoring of animal welfare at the individual and group levels. Automated facial analysis may detect pain, heat stress, aggression, disease, or environmental discomfort in pigs, cattle, sheep, poultry, and horses [39,43]. When integrated with edge computing, cloud platforms, and sensor networks, AI-based micro-expression systems could enable real-time alerts and welfare-informed management. At the governance level, such systems may contribute to digital animal welfare platforms by providing objective, continuous, and auditable indicators [38]. Nevertheless, implementation requires careful validation across breeds, housing systems, lighting conditions, and production environments.

Several studies illustrate the transition from concept to deployable prototypes. A pig heat-stress system combined an ASPP-enhanced YOLOv5 detector with facial-expression recognition [47], and sensor-based livestock frameworks have linked visual monitoring with thermal, wearable, and environmental measurements [43]. These examples support on-farm screening, but most remain single-site or proof-of-concept evaluations rather than fully prospective commercial deployments. At the infrastructure level, cross-domain AIoT research has shown how temporal prediction, privacy protection, and task offloading can support time-sensitive inference on resource-constrained edge networks [69]. Similar edge-cloud partitioning could reduce farm bandwidth and latency, but it must be validated under animal-specific camera, welfare, and data-governance requirements.

#### 3.4.5. Context-Specific Evidence: Laboratory Versus Companion Animals

Laboratory-animal and companion-animal applications require different evidence and deployment strategies. Laboratory studies typically use controlled illumination, fixed cameras, standardized stimuli, and experimentally defined or physiologically anchored labels, supporting reproducibility and causal analysis but limiting ecological validity. Companion-animal studies are conducted across clinics, shelters, or homes, where breeds, viewpoints, owner interactions, and environments vary substantially; labels often rely on clinician scores, owner reports, or contextual inference and may therefore be more subjective. Laboratory systems should emphasize preregistered protocols, cross-laboratory replication, and sensitivity to treatment or humane endpoints, whereas companion-animal systems should prioritize cross-breed/domain validation, uncertainty reporting, unobtrusive acquisition, and integration with veterinary judgment.

#### 3.4.6. Synthesis and Outlook on Translational Applications

Collectively, these translational applications represent a pivotal shift: AI-based micro-expression analysis is progressing from proof-of-concept to practical utility across laboratory, clinical, and agricultural settings. However, this transition remains incomplete. Most studies are still at the validation stage, with few demonstrating prospective real-world efficacy or cost-effectiveness. In basic research, the primary challenge is establishing causality—linking specific facial dynamics to underlying neural circuits and physiological pathways. For clinical and welfare applications, key obstacles include robustness across phenotypic variation and uncontrolled environments, standardization, and seamless integration into existing workflows.

Looking ahead, three strategic priorities emerge. First, validation must be prospective and outcome-driven, assessing whether AI-assisted monitoring improves clinical decisions, enables earlier interventions, or delivers measurable welfare benefits. Second, models should prioritize generalizability and uncertainty quantification over incremental gains in accuracy. Third, ethical and governance frameworks must evolve alongside technological advancements, addressing data ownership, privacy, and algorithmic bias through multi-stakeholder dialogue. Ultimately, the success of this field will be measured not by technical sophistication but by its contribution to more objective, timely, and welfare-centered animal care.

**Table 1:** Representative studies of AI-assisted animal facial and micro-expression analysis.

Species/Setting	Target State or Task	Input	Representative Approach	Main Contribution and Current Limitation	Ref.
Macaque	Affective-state recognition	Facial images/video	Automatic facial-expression classifier	Demonstrates automated primate state recognition; evidence remains controlled and species-specific.	[7]
Horse	Brief social facial movements	High-temporal-resolution video/FACS	Frame-resolved action-unit analysis	Characterizes subsecond facial signals; contexts are limited and it is not yet a general state decoder.	[9]
Mouse	Emotion states and neural correlates	Facial video plus neural recordings	Data-driven facial dynamics with neural association	Links facial configurations to neural state representations; conducted in a controlled, head-fixed setting.	[20]
Mouse	Well-being and pain surveillance	Facial images/video	Deep CNN	Supports automated laboratory monitoring; cross-laboratory and external validation remain limited.	[36]
Mouse	Multiple emotional states	Head-fixed facial images	Deep image classifier	Distinguishes experimentally induced states; acquisition and labels are highly constrained.	[37]
Horse	Pain recognition	Facial images/video	Deep-learning classifier	Supports objective pain-related facial assessment; generalizability across datasets and settings remains uncertain.	[45]
Pig	Heat stress	Farm facial images	ASPP-YOLOv5 plus classification	Provides a real-time-oriented prototype; multi-farm and cross-breed validation are still needed.	[47]
Dog	Tracking and emotion states	Surveillance video	CNN detection, tracking, and classification	Integrates monitoring with state recognition; labels are coarse and performance is environment-sensitive.	[48]
Dog	Anticipation and frustration	Facial images/video	Explainable classifier	Links predictions to interpretable facial regions; controlled elicitation limits transfer.	[49]
Cat	Pain	Facial images	Deep-learning classifier	Demonstrates automated feline pain recognition; viewpoint and clinical-domain shifts remain concerns.	[13]
Cat	Pain dynamics	Facial video plus landmarks	Landmark-based temporal classifier	Improves anatomical interpretability; occlusion and external clinical validation remain challenges.	[54]
Dogs/cats	Facial geometry, emotion, and pain	Images/video plus landmarks	Geometric morphometrics and landmark models	Provides interpretable species-specific quantification; definitions do not transfer directly across breeds or species.	[33,51,52,53]

**Table 2:** Critical comparison of major AI method families for animal micro-expression analysis.

Method Family	Representative Algorithms	Key Strengths	Main Limitations and Species Sensitivity	Small-Label Suitability	Best-Fit Scenario
Image classification	CNN, ResNet, EfficientNet, ViT, YOLO [30,37,47,48,49]	Simple end-to-end training; scalable; lightweight models can approach real-time inference.	May learn coat, background, or lighting artifacts; static frames omit defining dynamics; high domain-shift sensitivity.	Moderate with pretraining, augmentation, and subject-exclusive validation.	Fixed-camera screening, triage, and edge inference.
Landmark/pose	Keypoint detectors, geometric morphometrics, AnyFace++ [33,34,50,51,52,53,54,55]	Anatomically interpretable and compact; compatible with FACS/action-unit reasoning.	Requires keypoint annotation; vulnerable to occlusion; landmark definitions vary by species and breed.	Moderate to high when anatomy is standardized and pretrained detectors are available.	Pain scoring, FACS quantification, and controlled or semi-controlled views.
Temporal modeling	CNN-LSTM, TCN, 3D CNN, SlowFast, TimeSformer, VideoMAE [18,56,57,58,59,60,61,62]	Captures onset-apex-offset dynamics and longer behavioral context.	Needs video and computation; sensitive to frame rate, sequence length, subject leakage, and site shift.	Low without pretraining; moderate with self-supervision and regularization.	High-speed clips and longitudinal laboratory or clinical monitoring.
Multimodal fusion	Early concatenation, gated/attention fusion, and decision fusion [20,40,43,63]	Improves state disambiguation and links predictions to physiological or contextual evidence.	Requires synchronization and additional sensors; vulnerable to missing modalities; no consensus animal benchmark.	Low to moderate; late fusion is more tolerant of incomplete modalities.	Mechanistic laboratory studies, multimodal clinics, and precision livestock systems.
Foundation, VLM, and generative AI	Pretrained animal encoders, vision-language models, LVLM prompting, and synthetic-data generators [65,66,67,68]	Reusable representations; zero-/few-shot transfer; language-guided querying; augmentation of rare conditions.	Limited animal-affect evidence; context and anthropomorphic bias; hallucination; anatomical or synthetic-to-real mismatch.	Potentially high after species-specific adaptation; zero-shot outputs still require independent validation.	Exploratory transfer, annotation support, low-label pretraining, and anatomically screened augmentation.

## 4. Discussion

This review synthesizes a rapidly evolving field, demonstrating that AI-based decoding of animal micro-expressions has made significant technical advancements but remains in the early stages of broad translational validation. Our findings offer a comprehensive overview of current achievements and ongoing challenges. In this discussion, we contextualize our results within the broader literature, critically examine the limitations of the existing evidence base, and propose a strategic roadmap to advance the field.

### 4.1. Placing Animal Micro-Expression Decoding Within the Broader Landscape of AI-Based Behavior Analysis

To appreciate the current state and trajectory of AI-based animal micro-expression decoding, it is helpful to position it within the broader landscape of computational behavior analysis, particularly in relation to: (i) the well-established field of human micro-expression recognition, and (ii) the wider domain of automated animal behavior analysis.

**Comparison with Human Micro-Expression Recognition.** The study of human micro-expressions has a longer history, larger annotated datasets (e.g., CASME [70,71], SMIC [72], SAMM [73]), and well-established benchmarks that have driven rapid progress in deep learning architectures [74,75]. Moreover, human expressions can be validated against self-reported emotional states, physiological measurements, and social context, providing a relatively reliable ground truth. Animal micro-expression analysis inherits many of the same technical building blocks, but the transfer is far from straightforward. The absence of a verbalizable ground truth necessitates reliance on indirect proxies such as stimulus conditions, physiological correlates, or observer-rated pain scores [22], introducing greater label noise and ambiguity that limit the effectiveness of supervised learning. Furthermore, morphological diversity across species—varying fur patterns, ear shapes, muzzle structures, and facial mobility—renders generic face alignment and landmark detection models brittle; even within the same species, breed-specific craniofacial variations can drastically alter the appearance of facial actions [51,52]. Consequently, models pre-trained on human faces often require substantial domain adaptation, fine-tuning, or even complete re-architecture to perform adequately on animal data.

Despite these challenges, several aspects of human literature offer valuable pathways for animal research. Techniques for handling small sample sizes, such as transfer learning, data augmentation, and synthetic data generation, are directly applicable and have already been employed in canine and feline studies. The concept of explainable AI (XAI), which links model predictions to anatomically meaningful features like action units or landmark displacements, has been adopted to enhance interpretability in animal models [49]. Similarly, temporal modeling approaches have been successfully adapted to capture the dynamic evolution of animal facial expressions [18]. Moving forward, a more systematic cross-species transfer learning strategy, leveraging large-scale pre-trained models on human faces and fine-tuning them with species-specific data, may offer a pragmatic route to improve performance in data-scarce animal domains.

From a transfer-design perspective, generic visual backbones, optical-flow descriptors, attention mechanisms, self-supervised objectives, augmentation procedures, and subject-independent validation protocols can be transferred from human micro-expression research. Face detection, alignment, temporal windowing, and pretrained features generally require domain adaptation or species-specific fine-tuning. Landmark definitions, action-unit inventories, label ontologies, and ground-truth construction often need redesign because mobile ears, whiskers, muzzles, fur, nonfrontal head poses, and the absence of verbal self-report alter both the signal and its interpretation. A practical architecture is therefore a shared low-level encoder routed through animal-group-aware pathways and coupled to species-adapted anatomical heads and state-calibration modules, rather than a single unmodified human model.

**Comparison with Broader Animal Behavior Analysis.** While facial analysis focuses on a localized, information-rich area of the body, the field of automated animal behavior analysis has traditionally concentrated on whole-body pose estimation, trajectory tracking, and action recognition from video or sensor data. These approaches, often based on deep learning models such as DeepLabCut [76,77] and SLEAP [78], have revolutionized the quantification of locomotion and social interaction of animals [79,80]. Comparing these paradigms reveals both distinct challenges and synergies. Micro-expressions are more subtle, transient, and spatially localized than whole-body movements, requiring higher spatiotemporal resolution and more nuanced feature representation. Their interpretation is highly context-dependent—a slight ear movement or eyelid closure may carry different meanings depending on posture, social partner, or preceding events [10,28]. Conversely, facial signals can appear earlier than gross postural changes, offering potential early warning indicators [3].

Crucially, integrating facial and whole-body analysis can be mutually enriching. Whole-body behavior can serve as validation context—if a pain-related facial expression coincides with reduced mobility or guarding, the combined evidence strengthens state inference [22]. Facial and body features can be fused within multimodal models to improve robustness and provide a more holistic behavioral assessment [40]. Moreover, both streams share common infrastructural challenges—data scarcity, annotation burden, domain shift—and can benefit from shared solutions, including standardized protocols, open-source benchmark datasets with both facial and whole-body annotations, and cross-trained models that leverage complementary information. Recognizing these interconnections points toward a unified, multi-scale approach to automated behavior quantification.

### 4.2. Limitations of the Current Evidence Base

While the potential is immense, a critical assessment of the literature reveals several key limitations that temper our conclusions and define the frontier of future work. These limitations arise from both methodological constraints and inherent biological complexities.

**Data Scarcity and Species Imbalance**. The field faces significant challenges due to data scarcity and taxonomic bias. Studies on dogs, laboratory mice, and horses are relatively well-represented because of their close association with human contexts, whereas research on cats, pigs, cattle, sheep, and most non-human primates is comparatively sparse. This imbalance skews methodological development toward species with more human-readable and easily annotatable facial features, which may not serve as appropriate models for all research questions [4].

**The Ground-Truth Problem and Annotation Bottleneck**. Establishing reliable ground truth for internal states is inherently more challenging in animals than in humans. Researchers rely on indirect proxies—such as stimulus conditions, physiological correlates, or observer-rated scores—all of which are subject to confounds and only imperfectly correlated with subjective experience [22]. Annotation itself is labor-intensive, requires extensive training, and achieving inter-rater reliability for subtle movements remains challenging. The finding that canine facial expressions are modulated by human attention raises the concerns that observer presence may systematically bias measurements toward human-influenced expressions rather than authentic animal states [27].

**Limited Generalizability and Overfitting**. Most models are trained and tested on small, homogeneous datasets from single institutions, often under specific lighting conditions, camera angles, and with a limited number of subjects. This increases the risk of overfitting to dataset-specific artifacts. Consequently, the reported high classification accuracies are likely inflated. Few studies rigorously evaluate model performance on truly independent, multi-site, or cross-breed datasets, which is a minimum requirement for practical deployment [26,49].

Label uncertainty is not merely an annotation inconvenience. In supervised settings, inconsistent proxy labels can encourage models to fit annotator-, context-, or dataset-specific correlations, amplifying overfitting and failure under species, breed, site, or camera shift. Weak supervision can combine multiple imperfect sources—such as stimulus context, FACS/action-unit codes, physiological signals, expert ratings, and temporal heuristics—rather than treating any single proxy as definitive; behavior-analysis frameworks have shown that expert-informed labeling functions can reduce annotation demand [81]. Self-supervised learning can pretrain representations from unlabeled species-specific video before calibration with a smaller set of quality-controlled labels, as demonstrated in animal-behavior analysis by Selfee [82]. These approaches reduce dependence on noisy labels but do not resolve ambiguity in internal-state ground truth, so subject-exclusive and external validation remain essential.

### 4.3. Implications and Future Directions

These limitations, however, should not be viewed as insurmountable obstacles but rather as defining challenges for the next phase of research. To address them, we propose a series of concrete priorities:

**Building Open, Large-Scale, and Multi-Modal Benchmark Datasets**. The highest priority is the development of publicly available, large-scale, richly annotated datasets that encompass multiple species, breeds, ages, and environments, while incorporating multi-modal data such as video, physiological signals, and behavioral annotations. Standardized annotation protocols are essential for data harmonization. The “Animal Face” dataset series represents a promising step but requires significant expansion in both taxonomic diversity and contextual scope.

**Prioritizing Robustness and Generalizability.** Methodological development must shift from incremental benchmark improvements toward techniques that enhance robustness, including: (a) domain adaptation and generalization algorithms; (b) self-supervised, weakly supervised, and semi-supervised learning that leverage unlabeled video data; and (c) uncertainty quantification to identify ambiguous or out-of-distribution inputs, rather than producing overconfident predictions.

Data-efficient model development should combine four complementary strategies. First, foundation models and pretrained animal encoders can provide reusable visual or pose representations; SuperAnimal demonstrates that pooled multi-species data and standardized keypoints can support zero-shot or low-label transfer in animal pose analysis [65]. Second, supervised transfer learning and domain adaptation can reuse human, multi-species, or large generic vision representations while explicitly correcting species and camera shifts. Third, self-supervised learning can exploit unlabeled video through masked reconstruction and temporal-consistency objectives before limited task-specific fine-tuning. Fourth, synthetic images or videos can expand rare poses and states, but generated samples must be screened for anatomical plausibility and evaluated only on independent real data. Adaptive online-learning frameworks from other domains also illustrate how models can update representations as contexts evolve [83]; their use in animal micro-expression decoding remains a future research question rather than established evidence.

**Closing the Translational Gap**. Prospective, controlled translational trials are urgently needed to evaluate AI-assisted assessment in real-world settings such as veterinary clinics, commercial farms, and research facilities. These studies should measure practical outcomes, including time savings, impact on clinical decisions, user acceptance, false alarm rates, and tangible improvements in animal welfare. Additionally, failure analysis and the reporting of negative results should be actively encouraged.

**Figure 3:**
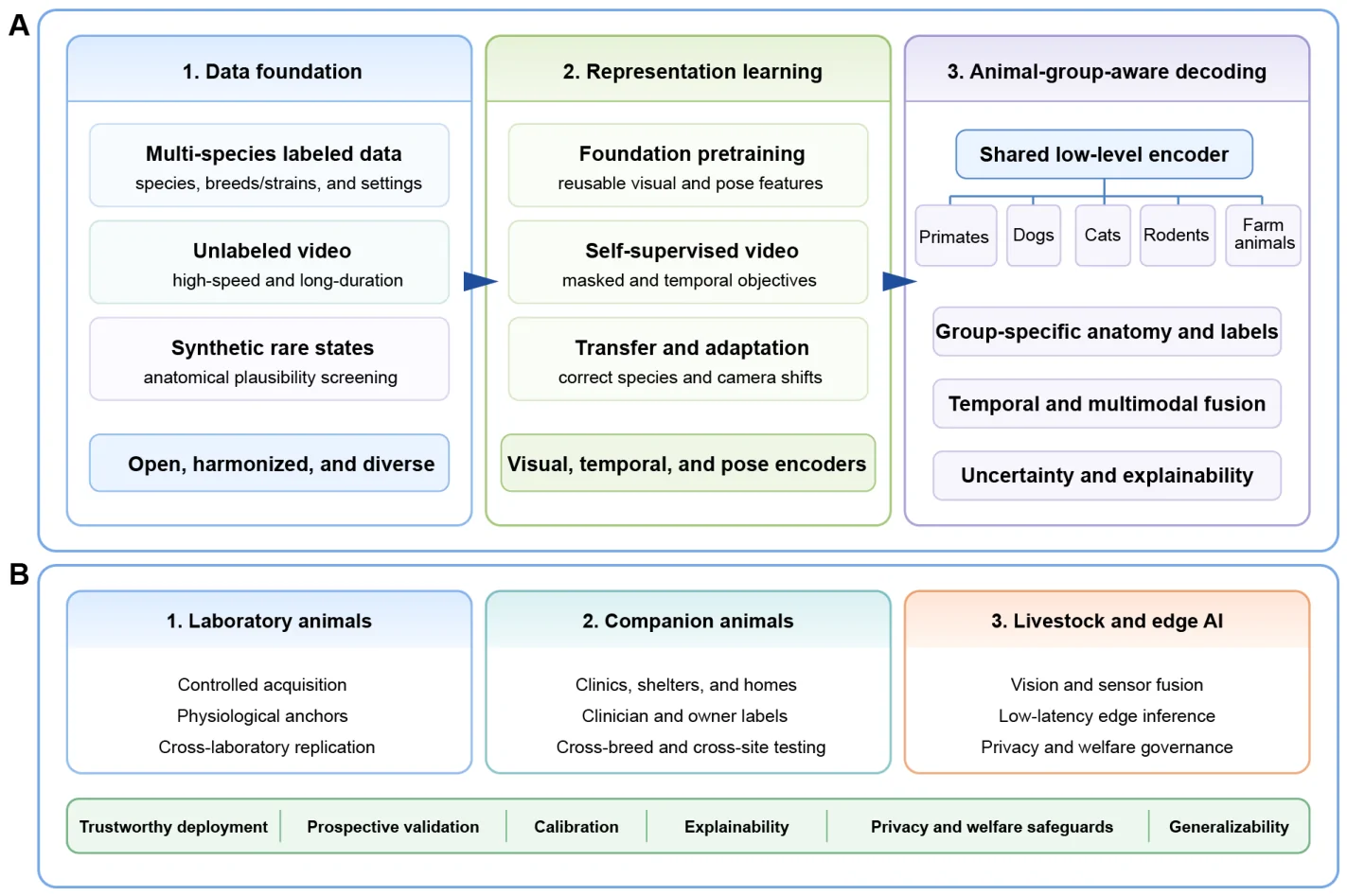
**Roadmap for data-efficient, species-aware development and context-specific validation of AI-based animal micro-expression decoding.** (**A**) Open, harmonized multi-species data, unlabeled high-speed video, and anatomically screened synthetic samples support foundation pretraining, self-supervised video learning, and transfer/domain adaptation. Shared low-level representations are routed through five high-level animal-group pathways matching the categories in Figure 1: primates, dogs, cats, rodents, and farm animals. The five groups provide a consistent organizational framework across the figures. They do not imply a single anatomical or label space within each group; individual implementations may still require species- or breed-specific anatomical representations, label systems, and calibration. The resulting models integrate temporal and multimodal information and support uncertainty-aware, explainable outputs. (**B**) Validation and deployment priorities differ across laboratory animals, companion animals, and livestock/edge-AI settings. Prospective validation, calibration, explainability, privacy and welfare safeguards, and generalizability are common requirements for trustworthy deployment.

In summary, AI-based decoding of animal micro-expressions is at a critical juncture. While core technical components are sufficiently mature to support prototype development, overcoming fundamental limitations in data availability, validation processes, and interdisciplinary collaboration is essential for transitioning to routine and reliable animal assessment. The proposed data-efficient, species-aware development and context-specific validation roadmap is summarized in Figure 3.

## 5. Conclusions

In conclusion, animal micro-expressions represent a rich yet underutilized behavioral modality that provides a unique window into the internal states of non-human animals. This review demonstrates that AI-based methods collectively offer a powerful technical foundation for transforming these fleeting and subtle signals into quantifiable, objective, and interpretable behavioral biomarkers.

However, the field is at a pivotal inflection point. The transition from proof-of-concept accuracy in controlled laboratory settings to reliable, real-world utility presents a formidable yet surmountable set of challenges. The most pressing priorities for the next phase of research are clear: (1) Build the data infrastructure by establishing large-scale, multi-species benchmark datasets with rigorous, harmonized annotation standards; (2) Develop robust and trustworthy AI models that prioritize generalizability, interpretability, and uncertainty quantification over incremental performance gains on existing benchmarks; (3) Demonstrate translational value through prospective validation studies that evaluate AI-assisted assessment against clinically and operationally meaningful endpoints, rather than solely retrospective accuracy; (4) Foster deep interdisciplinary collaboration to ensure that technical development is not only technologically sound but also biologically meaningful and directly aligned with practical needs in veterinary medicine, biomedical research, and animal welfare governance.

With sustained, coordinated investment, AI-based micro-expression decoding can evolve from a promising research methodology into a standard component of objective, scalable, and humane animal assessment, transforming how we understand, care for, and interact with animals.

## Data Availability

No new data were created or analyzed in this study. Data sharing is not applicable to this article.
